# Bibliographical Notices

**Published:** 1854-03

**Authors:** 


### BIBLIOGRAPHICAL NOTICES.

*Homœopathy. Its Tenets and Tendencies, &c.* By James Y. Sympson, M. D., F. R. S. E., Professor of Midwifery in the University of Edinburgh, &c. Lindsay & Blakiston: Philadelphia.

*Homœopathy Fairly Represented—in Reply to Dr. Simpson's, "Homœopathy" Misrepresented.* By Wm. Henderson, M. D., Professor of General Pathology in the University of Edinburgh.

The claims of Homœopathy upon our attention are of such a nature, both as regards its theory and the influence which that theory has exerted and still exerts upon the public, that false, unphilosophic, and, as a practice, negative as we may deem it, it fairly demands at our hands a respectful investigation. Ridicule and obloquy do not comport, in our view, with the consideration due to so important a subject, and we may be sure, that their indulgence, however tempting, must materially retard that final judgment, which all should be anxious to arrive at. Entertaining these opinions, we shall endeavor to give our readers a fair and candid notice of the above works; with the impartiality of an honest critic, neither withholding censure where deserved, nor refusing to recognize the truth wherever we may find it.

The acknowledged talents of the two writers, both of them Professors in the University of Edinburgh, are, in a measure, guarantees for the able manner in which they have respectively treated their subjects. A very different spirit however animates and breathes throughout their works. Dr. Simpson always preserves his good temper. Dr. Henderson, on the contrary, exhibits a rankling bitterness of feeling which sets all courtesy at defiance.

Both writers introduce too much irrelevant matter. In this particular Dr. S., as the aggressor in the contest, is most deserving of blame. We will only mention two instances: the story about the box of homœopathic globules lent to Dr. H., and Dr. Horace Green's relation of certain incidents. Those who have perused the reply, will agree with us that nothing is gained by the introduction of these matters. The affair of the box is related differently; in fact, they are at direct issue upon it. As for our unfortunate countryman, Dr. Green, from his entrance on the scene until his dismissal, he is fairly pilloried for the reader's amusement; his suggestive name causing to be showered down upon him the most merciless and contemptuous ridicule—in our opinion, alike unwarrantable and ungentlemanly.

This clap-trap, theatrical style of writing is much indulged in by Dr. H.; when hard pressed he always turns to it, like the fox to his cover.

We are constrained to say that we cannot agree with Dr. Simpson in his condemnatory remarks of the psoric hypothesis, commonly called the itch doctrine. His mention of this, as an instance of Hahnemann's folly and ignorance, affords Dr. H., who, it will be remembered, is Professor of Pathology, an excellent opportunity of reading him a lesson, of which he eagerly and fully avails himself. As the subject is quite interesting, we shall afford our readers an opportunity of forming their own conclusions. "Psora," says Dr. H., "was an ancient term used almost indiscriminately for every diversity of chronic and almost every kind of acute cutaneous disease." "The discrimination of a *particular disease* which should be distinguished as *itch* from all other skin diseases, by its *insect* (or rather arachnid) more especially, is entirely a modern accomplishment, indeed, as a general attainment, it is but a very few years old, and *was not recognized at all by Hahnemann.*" Dr. H. states that Hoffmann, among the many "other diseases" which he ascribes to the itch (meaning by that term any chronic skin disease) mentions "epilepsy, amaurosis, hematuria, consumption, rickets, hooping cough, apoplexy, and rheumatism." In our own times, Schönlein the able Professor of Pathology and Therapeutics in the University of Berlin, strongly advocates the same view. Mr. Paget and Dr. W. Budd are also adduced as supporters "of the doctrine that a morbid material in the blood is the cause of many diseases of the skin, bones, joints, arteries, &c., not always ascribed to such a source." Dr. Budd, after expressing his opinion that this morbid matter produces skin diseases by entering into union with portions of the cutaneous tissue, says "that the morbid matter is liable from various causes to be repelled from the surface, and in consequence to produce numerous disorders in internal parts." "Without believing," says Dr. H., "that the modern doctrinal pathology of the allopathic physicians to whom I have referred is correct in every particular, I am prepared to contend that it is just in the main, and *must* be held by every well-informed and observant physician. For Hahnemann's doctrine differs in no one essential particular from theirs, and has no more to do with the itch than theirs, nay, much less; for Schönlein specifies the *particular disease*, now distinguished as *itch* by its insect, as capable of causing internal diseases, which Hahnemann *never did.*" The gauntlet is then thrown down by Dr. H. before his adversary, and he is challenged to take it up and argue the point in issue, for the honor of his sect, if he believes them to be committed to the rejection of this hypothesis.

If the above be their belief, we sincerely hope that hereafter we shall be spared any further ridicule of this *itch-doctrine.* It seems to us to be in entire consonance with the humoral pathology of the present day. Whether this be so or not, however, it has no connection whatever with either the theory or practice of Homœopathy, and we can see no good reason why it should be always dragged into the contest.

We shall now consider the real points at issue, the Theory and Practice of Homœopathy.

The universal application of the doctrine,—*similia similibus curantur*,—which means, that to effect a cure we must prescribe a remedy which can itself create an affection similar to that sought to be cured; the provings of these medicines, which are the effects they produce on persons in health, or the new symptoms they cause to appear in the sick; and the infinitisimal doses in which these latter are given, are the essential characteristics of Homœopathy. These are separately and we think fairly and candidly examined by Dr. Simpson.

The first, claimed by Hahnemann and his followers to be the only universal, infallible therapeutic law, is shown to exhibit none of the characters of a law, even of a low degree of generality, much less those of an universal one. "It would be a very wearisome," Dr. Simpson observes, "and a very useless task to follow out this enquiry in relation to any great number of diseases. Let us consider it in its bearings upon two or three only; for, in truth, our limited space forbids us to allude to more. And for illustrations, let us take the examples which Sir John Herschell adduces in his "Preliminary Discourse on the Study of Natural Philosophy," as instances for which the advancement of Science, of late years, has devised more or less certain means of removal and cure. These instances were, of course, originally cited by that author without any view to such a question as the present; and are hence free from the prejudice of selection. Sir John Herschell adduces as his illustrations the treatment of the following four diseases, viz: the cure of ague, or intermittent fever, by quinine; the arrestment of the ravages of small pox, by vaccination; the suppression of sea scurvy in our fleets, by lemon juice, &c.; and the removal of goitres, by the use of iodine. These examples are as good as any other, and for the present object the more apposite, inasmuch as the first in our enumeration formed in Hahnemann's mind tho alleged fact by which the doctrine of homœopathy was primarily suggested to him; and the second has been adduced by his disciples as the best marked instance in favor of his doctrine."

Hahnemann supposed that cinchona cured intermittent fever, because, when taken in ordinary doses, it produces, in the healthy system, an intermittent fever, or symptoms similar to it. "Like cures like." In regard to this statement, it is observed by Dr. Simpson, that "there is scarcely a British medical practitioner who has not exhibited cinchona or quinine to his patients hundreds of times. Among our population many thousands of individuals have taken the cinchona, or its concentrated principle, quinine, and the alleged result, viz., the production of ague, has many thousands of times failed. Indeed, most of the individuals in these islands who have been repeatedly sick (and who has not?) have taken, at one time or another, doses of cinchona or quinine as a tonic or otherwise. Has one of them ever had ague, or a disease like ague, produced by the use of this drug? I am informed that one house in London manufactures every year, for medical uses, about £30,000 worth of quinine. Does a single dose out of this vast quantity ever create ague in those to whom it is administered?" "Besides, many people have taken continuous doses of quinine to acquire increased tone and strength, even after they were in a state of comparatively good health; and not a few more of our officers and others serving on the coast of Africa, and in India, have (as was first, I believe, recommended by Dr. Copeland) taken long courses of quinine without observing ague to follow its use."

In regard to vaccination and its effects upon small pox, Dr. S. states that, "in order to cure a disease by means of a similar affection developed by a homœopathically chosen remedy, we should, according to Hahnemann, excite in the body an artificial or 'medicinal disease somewhat greater in degree' than the natural disease which we wish to remove. (Organon, P. 41.) The physician, he elsewhere observes, 'affects the vital force in a stronger manner by a potency that produces a disease very similar but *stronger*.'" (P. 128.) "When, however, we use, as a preventive of small pox, artificial inoculation with small pox, (variolation), or artificial inoculation with cow pox (vaccination), the artificial disease is not, what is hypothetically required by the homœopathic law, a *greater or stronger* disease than the small-pox itself. Besides, when small-pox inoculation was used, as was very generally and very successfully done in the last century, to prevent small pox, (and has been lately proposed to be done again by some modern physicians, as Drs. Copland, Gregory, and Trousseau,) the remedy thus employed did not pro duce symptoms merely *similar* to small-pox, but symptoms *identical* with it. In fact the two diseases were identical and the same. The effect, if explicable in this way at all, was not certainly an effect of the homœopathic law, similia similibus curantur,—of like cured by like; but rather of the isopathic law of Luz, viz., of the same disease cured by the same,—æqualia æqualibus curantur. And the same remark may be extended to the present practice of vaccination, seeing that almost all pathologists are now agreed that small-pox and cow-pox are identically the same disease, with this difference, that the cow-pox has been modified by passing through the body of the cow, but can be reproduced again in the cow by exposing it fully to the contagion of human small-pox."

*Prevention and Cure of Sea-Scurvy by Lemon-juice.*—"Scurvy," says Dr. Watson, "is infallibly and rapidly cured by the administration of lemon-juice, or of other fresh fruits and vegetables." "But again," says our author, "while the use of fresh and succulent vegetables, such as salads, fruits, etc., will almost infallibly *cure* any common case of scurvy, certainly the daily use of salads or fruits by the general community does not *produce* in them that disease; and yet, I repeat, such a diet ought to have such an effect, provided there were any truth in what Hahnemann terms his "infallible, eternal law of nature."

*Cure of Goitre by the Exhibition of Iodine.*—"In consonance with the unerring law of homœopathy, this drug, which thus removes goitrous swelling of the neck, ought also to produce an affection similar to goitre in the neck or thyroid gland. But though preparations of iodine are used most extensively in medical practice for other diseases, (about 50,000 lbs. of iodine being manufactured annually in Great Britain for medical purposes alone,) no one, I believe, has ever seen the drug produce this affection, or any affection in any degree similar to it, in the neck or in the thyroid gland, which it ought constantly to do, under these circumstances, if there were truth in the homœopathic law. We are told, however, by the homœopathists, that some day they expect to see an instance in which the use of iodine will coincide with an enlargement of the thyroid gland, or with symptoms of goitre. And they agree that this "coming" case will prove that the action of iodine is homœopathic; illogically arguing in this, as in other instances, that any such rare exception, provided it ever did occur, ought to be received as a general and universal fact. As well, and as logically, might a naturalist take it upon him to aver, as a general law, that man was actually a treble-headed animal, and, in confirmation of it, tell us that he was merely waiting for an instance of the birth of such a human monstrosity showing three heads, believing that such an occurrence would prove the truth of his supposed generalisation." These extracts are sufficient to satisfy any intelligent mind, that the law of similia similibus curantur, does not hold good as regards the remedies above mentioned. And as they were not selected by the author, we think it but reasonable to infer that it is equally inapplicable to the remainder of our materia medica.

The special symptoms produced by medicines are obtained by Homœopathists from several sources. These are the known effects of special remedies in particular diseases; the symptoms exerted by the poisons on the healthy; and finally, and principally, the effects or "artificial symptoms" which follow the exhibition of their various drugs upon persons in health or in a state of disease. These latter are called "provings." Common salt, *proved*, causes 450 symptoms, and consequently cures them when occurring in a state of disease. It is usually employed in doses of the twentieth or thirtieth dilution, and the effect of one of these is said to continue, in chronic affections, from 40 to 50 days. Chalk employed in the same dilution causes 1000 symptoms. The duration of *its* effects are fifty days in chronic diseases, and so on. Some of the symptoms recorded by Jahr, in his Materia Medica, as occasioned by different drugs, are most extraordinary and unnatural. Salt, besides other symptoms, causes paralysis; tendency to experience dislocation; typhus fever with debility; hatred to persons who have formerly given offence; numbness on one side of the nose; disagreeable risings after partaking of fat food or milk; excoriation between the buttocks, especially when walking; numerous flaws in the nails; redness of the great toe; corns on the feet. Chalk causes a strong desire to be magnetized; emaciation without failure of appetite; great plumpness and excessive obesity; flaws in the fingers; snoring during sleep; quotidian fever towards two o'clock in the afternoon, or tertian fever in the evening; dizziness after scratching behind the ear; immense size of the head; lachrymal fistula; dilatation of pupils; polypus in the ears; fœtid smell from the nose; before the evacuation, great irascibility; warts on the arms and hands, &c. Sweet violet causes an aversion to all kinds of music, and principally to the violin; the symptoms of doses of colchicum are singularly aggravated by a too brilliant light, and by the smell of pork; sulphur causes easy dislocation of the foot when walking. The provings of two hundred other drugs, as recorded by Jahr, are quite as absurd as those we have mentioned. Some of the delusions caused or said to be caused by certain medicines are quite curious. Thus the delusion that he is riding an ox is produced by belladonna; that he is a goose, by conium; that he is a child, by cicuta; that he has old chairs to mend, by copper; that his head and nose are transparent, by belladonna; that his feet are in his brain, by amphisbœna; that men are swine, by henbane; pretending to crack nuts, by the same; pretending to drive away peacocks, by hyoscyamus; eats his shoes, by veratrum; tries to climb up the stove by henbane, &c.

Surely any comment on the above is unnecessary.

There are four "doses," "potencies," "divisions," "dilutions," &c., in use among homœopathists. The lower range from the original form of drugs up to the sixth attenuation. The middle from the sixth to the thirtieth. The higher from the thirtieth to the two hundredth, and the highest from the two hundredth to any where above that number. Several homœopathic works give their readers a standard table, showing the amount of a grain or drop of any drug in the "lower" or "middle" dilution. "The first is one hundredth of a drop or grain; the sixth is one billionth; the twelth one quadrillionth; the eighteenth one sextillionth; the twenty-fourth one octillionth; the thirtieth one decillionth, or 1,000,000,000,000,000,000,000,000,000,000,000,000,000,000,000,000,000,000,000,000,000th. This decillionth, in other words, "consists of a minute globule of sugar, moistened by being simply dipped in a drop out of an ocean of fluid one hundred and forty billions (or 140,000,000,000,000) times as large as our whole planetary system, and which enormous ocean has been medicated for the purposes of homœopathy, by having dissolved and mixed through it one single grain of the appropriate drug."

Such are the astounding results obtained by calculation. As a pledge for their accuracy, (and for many others in the appendix,) we are told in the Preface that they have been carefully looked over by four or five of the most distinguished mathematicians in Great Britain.

How does homœopathy fairly represented answer these matters? How is its author enabled to stand before such a storm of facts, figures, illustrations and arguments, as assail him in Dr. S.'s book, capable, one would think, of crushing a far more defensible theory into the most infinitesmal atoms? We will inform our readers. Like a skilful counsel, whose case is considered perfectly desperate by all who have listened to the testimony, Dr. Henderson commences his reply by boldly assuming, not from any "vain confidence" in his own powers, but entirely from the weakness of his adversary's argument, that the verdict must be given in his favor. Then discreetly yielding, with a charming show of candor, what would have been folly in him to have attempted to maintain; by availing himself of every assailable point of his adversary's reasoning, and slighting his strongest and most important arguments; by charges of misrepresentation, cries of persecution, threats of exposure, and contemptuous flings at the testimony and witnesses adduced, he hopes, in the dust, "noise and confusion" thus raised, to conceal his discomfiture and defeat. Less arrogance and presumption, while it would not have detracted from his arguments, would, at least, have secured for him more of the respect and sympathy which should always be accorded to the vanquished.

The points at issue, as he himself states, are "partly about the use of the law, partly about the provings, and partly about the dose." Let us hear his defence. And first on the universal application of the law—similia similibus curantur. It will be remembered that four instances, which we might almost say were taken at random, were adduced by Dr. Simpson of the inapplicability of this law. To the first of these, the cure of ague by quinine or cinchona bark, which should consequently cause ague, he rejoins that such is its effect. Two of the authorities he quotes to prove this, Pereira and Christison, respectively mention that by large doses "a febrile state is set up, (manifested by the excitement of the vascular system and dry tongue,)" and that "it is apt to excite nausea, pain in the stomach and *febrile symptoms.*" The other, Guersant, observes of the sulphate of quinia, that "it excites a true febrile movement of more or less duration;" and of the bark itself, he says, "the reaction which it manifests many hours after its reception is, in general, much more marked (than of the sulphate;) it manifests itself by heat of skin, by more vivacity and energy in the motions, &c., although the *febrile condition* is not long continued." Again, in regard to the sulphate, the same author mentions, that in some it produces "great anxiety, acsompanied with *shiverings*, faintness, *cold sweats* and agitation."

Referring our readers to Dr. Simpson's remarks on this point, we shall merely add, that neither of the above writers say one word about the *periodicity* of the symptoms they mention, which it is unnecessary for us to observe, is the peculiar characteristic of ague.

*Vaccination and its effects upon Small Pox.*—Dr. Henderson contends, that although inoculation of the cow with small pox virus produces the vaccine disease, that the two diseases are not identical, though similar; and that it is in virtue of this similarity that the vaccine virus is homœopathic to small pox. This is easily refuted. Vaccination, inoculation, (or what is the same thing, an attack of small pox,) are equally preventive of that disease. An attack of scarlet fever, or measles, or chicken pox, prevent in the same way *their* recurrence. Do each of these latter diseases, from its being homœopathic to itself, prevent this liability to a second attack? The truth is, that all these affections are non-recurrent, and the chance of a successful re-vaccination is about equal to that of a second attack of small pox.

As regards the prevention and cure of sea-scurvy by lemon-juice, we are informed that Dr. Stevens *has* related a case in which citric acid produced scurvy. Also, that as it is entirely due to unsuitable food, it is consequently cured by restoring to the diet the ingredient that is deficient. Thus potatoes, milk, &c., cure scurvy as well or better than any substitute given by physicians. Lemon-juice is said by some to act dietetically by supplying the potash salts, which are believed to be wanting. For these reasons, he is surprised that scurvy should be adduced as a case for drugs at all.

We are perfectly willing to waive the matter, as we think his objections are, in the main, just. If lemon-juice be dietetic in its action, it proves nothing for homœopathy. On the contrary, if it act medicinally, as supposed by most physicians, we ought to see, which we do not, cases of scurvy all around us, produced by its use. As regards Dr. Stevens' case, it is but reasonable to suppose, that as in all time and by no other writer is such a circumstance related, that he was incorrect in ascribing such a result to its use.

*Cure of Goitre by Iodine.*—Of this substance Dr. H. observes that if it "be not homœopathic in its action ('to scurvy') what is it? no one can tell, or at least *prove*, anything on the subject." Also, that for certain effects in the provings of drugs "to be characteristic, they need not be constantly produced, or even in many persons." Some approach to the pathogenetic effects in question, as glandular swellings in the neck and elsewhere, and the increase of the disease at the commencement of its exhibition, has been made in the provings of iodine. In a note appended, we are informed that "a man taking four grains of hydriodate of of potass twice a-day, became affected with a "rapidly growing swelling of the thyroid gland." See Brit. Jour. of Hom. No. xliv. We utterly deny these effects from the *use* of iodine. They are against all experience. That *during its use* such symptoms, and many others, have occured, is quite probable. "There are more false facts," as Cullen remarks, "than even false theories in medicine."

Even supposing that these remedies, quinine, iodine, and (from his relation of Dr. Steven's case,) lemon-juice, are homœopathic to the respective diseases in which they are given, which it is proved they are not; what, as they are always given in ordinary and sensible doses, not being curative when used billionthly, what, we would like to know, becomes of the solemn Hahnemannic law that, "It holds good and will continue to hold good as a homœopathic therapeutic maxim, not to be refuted by any experience in the world, that the best dose of the properly selected remedy is always the very smallest one in one of the high dynamisations ($\overline{X}$ or the 30th dilution,) as well for chronic as for acute diseases." (Organon, p. 289.) To which law, the Journal of Homœopathy for 1849, p. 452, states as the only exception, the administration of camphor-spirit in cholera.

As regards the "provings" Dr. Henderson observes that they are not, in reality, as numerous as they seem to be. Calcarea or chalk appears to produce 1090 symptoms. The real number is much less, probably not one fourth or one sixth. This is due to the fact that many of the symptoms are mentioned more than once, being split up, as it were, either by the peculiarities of the persons in whom they were produced, or by other causes, illness, &c.

Allowing 100 symptoms to each of their 200 *proved* drugs, gives them 20,000 symptoms. We ought to congratulate ourselves that legitimate medicine has not been so extravagant in this particular.

It is allowed that many of the symptoms enumerated are insignificant. "Hahnemann, more especially, committed atrocities of this kind, but it was an error, if error it was, on the safe side." As to the *moral* and *religious* symptoms and states produced, and consequently caused by certain medicines, as for instance, despair of eternal happiness, by pulsatilla; excessive scruples of conscience and despair of one's self and others, by gold; absence of all moral and religious feelings, by anacardium, &c.; he upholds them all, appealing to the experience of physicians, whether every kind of religious feeling may not depend upon mere bodily states. "How, then, can it be a matter of surprise to any man that medicines capable of disordering the body, the stomach as well as other organs, should also, and for that very reason, be capable of exciting or disordering the feelings of the mind? Does not alcohol do so? Do not opium, belladonna, hyoscyamus, 'hackisch,' and many more? And what medical man can deny that they do?"

This, though very plausible, is not at all pertinent to the matter. The dependence of the mind upon the body is not in question. Peculiar conditions of the mind, such, for instance, as absence of all religious feeling, and excessive scruples of conscience, exist, without question, *independently* of bodily disease. Yet, according to Jahr's Manual of Hom., lachesis causes and hence cures the former? and gold the latter. Shakspeare should have taught them better. When Macbeth, in reference to Lady Macbeth's condition, says to the Doctor, "Can'st thou not minister to a mind diseased"? &c., he is answered, "Therein the patient must minister to himself." If we believe the homœopathists, however, her terror and despair, the whips and stings of an awakened conscience, might all have been allayed, nay more, effaced by a billionth of a grain of gold or pulsatilla.

In answer to Dr. Simpson's remark, that chalk (carbonate of lime) and common salt are found, the former in most of the vegetables and in almost every water used by man, and the latter in our food as a universal condiment, we have given us four or five pages of comment, which exhibit a most unpardonable ignorance of chemistry on the part of Dr. H. Carbonate of lime, and the remark equally applies to any other salt, whether prescribed homœopathically or taken in our water and food, must always meet with the same acid in the stomach and be alike affected by it. As to salt, (chloride of sodium,) it is found in all of the secretions, urine, sweat, tears, saliva, &c. ; and it is perfectly preposterous to suppose that a billionth of it can act remedially, when we know that it is not only extensively used, but that a desire for it is instinctive in both man and beast.

The calculations, given us by Dr. Simpson, as to the real amount of medicinal matter existing in their infinitesimal doses, are sneered at but not disputed.

We have no desire to plume ourselves upon the victory gained by Dr. Simpson in this "battle of the books." Brilliant as are his adversary's talents, specious his arguments, no other result could have been expected. On the one side, legitimate medicine, with its accumulated stores of learning, based on physiology, pathology and therapeutics; on the other, a doctrine which abjures all past experience and all present science, and which taken in connexion with its practice is not only visionary and fantastic, but absolutely repulsive to common sense; a doctrine which, in the words of Dr. Forbes, "is at once false and bad, useless to the sufferer and degrading to the physician." Such in our belief is homœopathy.

There is great discrepancy of opinion regarding the character of Hahnemann. "It has often been questioned," observes Dr. Simpson, "whether Hahnemann, like the 'prophet' Joe Smith, used the talents bestowed on him only to dupe others, or whether he was enough of a visionary to believe in his own various incredible doctrines. The published evidence of one who knew Hahnemann, Dr. Schubert of Dramburg, goes far to prove that the founder of homœopathy was himself far more knavish than foolish." "On one occasion," says Dr. Schubert, "he said to me, 'I give medicines but very seldom, although I always prescribe small powders. I do this for the sake of keeping up in the patient's mind the firm belief that each powder contains a particular dose of medicine. Most patients will get well by adopting a simple mode of living, and by placing a boundless confidence in their medical attendant. Ordinary practitioners know nothing of this, practically, although they are always talking of the healing powers of nature.'" (Casper's Wochenschrift for 1845.) His disciples, on the other hand, speak of his 'long and noble life;' of his being 'a monument of truth, of learning, and of greatness;' of the 'eye that dwelt so long and intensely on the light that appeared amidst the general gloom,' &c.

Notwithstanding Dr. Simpson's statements above given, we rather incline to the opinion that he was a visionary. His belief in dynamization by friction and shaking as a means of developing medicinal force; his ascription of most of "the ills that flesh is heir to," first to the use of coffee, afterwards to psora; his discovery of wonderful and unheard of qualities in the most simple and extensively used substances; his strong faith in his 30th dilution, superseded as he grew older by the conviction that *olfaction* of a dried decillionth was equally powerful; all these enforced with an enthusiasm and blind confidence in his own powers and a most arrogant contempt for the opinions of others, demonstrate this peculiar mental condition.

Nor are Schubert's statements, nor the fact that Hahnemann advocated very opposite opinions and modes of treatment at different periods of his life, at all at variance with this idea. In truth these contradictions and inconsistencies belong to and are needed to complete such a character.

The doctrine of similia similibus curantur, it is well known, did not originate with Hahnemann; it having been promulgated and occasionally applied by many writers previous to his time. In the treatise by Hippocrates, "On the Places in Man," published by the Sydenham Society, p. 77, we find the following: "He then insists in strong terms, that under certain circumstances, purgatives will bind the bowels, and astringents loosen them.

And he further makes the important remark, that, although the general rule of treatment be 'contraria contrariis curantur,' the opposite one holds good in some cases—namely, 'similia similibus curantur.' " In Sir John Floyer's History of cold bathing, a very curious old work, published in 1722, we have it also remarked to the same effect: "and why sometimes they gave water warm (he says) because they supposed the distemper to proceed from a cold cause, so proceeded according to the axiom, contraria contrariis, &c., which is not always orthodox, for very often similia similibus sanantur."

We may mention also that Dr. Armstrong's first case (see his Life by John Boott, M. D.) was one in which he applied this principle correctly. It was a case of diarrhœa, of two years continuance, which had resisted all the skill of his medical advisers. Conceiving, from the history of the case, that it was one of overloaded bowels, and that the diarrhœa was merely the effect of the irritation thus established, he advised a mild course of laxatives. This produced the desired effect, and the patient was entirely restored to health.

As a general, much less a universal law, it has never, however, been recognised by the profession; and even its occasional application to practice may be explained on other grounds, in almost every instance.

In Dr. Ware's admirable Discourses on Medical Education we find the following prophecy, with which we shall conclude our notice: "Other systems will pass away,—ours will be permanent; nourished, indeed, by the very elements which come from their decay, as the eternal oak flourishes and grows green for ages from the decomposition of the transient vegetation, of which generations are springing up, and perishing around it."

---

*The Diseases of the Rectum.* By Richard Quain, F. R. S., Professor of Clinical Surgery in University College; Surgeon to University College Hospital. London: Walton & Maberly.

This book is made up of a series of clinical lectures delivered by Mr. Quain at University College, London. It is written in a plain, unaffected style, and sets forth with clearness and precision the symptoms and treatment of the different diseases which are met with at the lower part of the rectum. The observations are all based upon cases which have been under the writer's own care. These cases are well drawn up and illustrate fully the diseases on which the author treats.

The author has not attempted a systematic arrangement, his "object being not the study of the natural history of disease, but the history of patients, their ailments and their cure, the classification of the subjects of these lectures—beyond the grouping together those that present obvious affinities—is unimportant." We consider this a great defect in the book. If by "natural history of disease" the author means the origin, nature and consequences of morbid processes (and he can mean nothing else,) nothing can be more hurtful to scientific surgery than the neglect of such a study. The phenomena of disease, if carefully collated with each other, lead us, as it were by the hand, to those palpable indications of treatment which are drawn not from the hallucinations of our fancy, but from the innermost penetralia of nature, (Sydenham.) This holds good equally in surgery as in medicine. It is just this knowledge that puts the well educated surgeon above the charlatan and empiric.

We regret to be obliged to say, that in our opinion, Mr. Quain has not done justice to Professor Syme, of Edinburgh. To that eminent surgeon the profession in Great Britain is chiefly indebted for bringing before them the observations of M. Ribes respecting the situation of the internal orifice of *fistula in ano*, and for pointing out the inutility of cutting beyond that point. He it was, also, who first combated the then prevalent opinion which referred the origin of fistula to ulceration of the mucous membrane of the bowel; to him we are indebted for describing the true nature of polypi, fissures of the anus and stricture; and for founding upon their "natural history," *i. e.* their pathology, a simple and effective treatment.* These things were surely known to Mr. Quain; why were they not duly credited? The omission is the more observable as Mr. Quain is by no means *costive* in referring to the "doings" of London and Dublin surgeons.

* See Diseases of the Rectum, 1838. This work has reached a 3d edition.

We have no space for anything like a critical analysis of the book. We can sincerely recommend it as an excellent practical guide on those diseases of which it treats, although it must be confessed, as far as we have been able to see, it presents but little that is novel either in diagnosis or treatment.

---

*The Diseases of the Heart and the Aorta.* By WILLIAM STOKES, Regius Professor of Physic in the University of Dublin, &c. Lindsay & Blakiston, Philadelphia.

We do not propose to enter upon any formal notice of the above work. Dr. Stokes' experience, abilities and character are so highly appreciated both in Europe and in our own country, as almost to place his writings beyond the pale of criticism. Few physicians have had such rare opportunities for observing disease, and we may safely assert that none have better profited by them. Entertaining these opinions, which we believe to be universal with the profession, we deem any criticism of his work unnecessary, and shall, therefore, merely state its scope and nature, which embodies the results of "clinical observations, continued almost unremittingly for upwards of a quarter of a century."

In the Preface, we are informed that the work "is not intended as a full treatise on cardiac pathology, nor yet on physical diagnosis, but it aims at the rational application of these branches of knowledge to practical medicine." "The diagnosis of the combination of diseases even in so small an organ as the heart is still to be worked out, and until this be done, the rules of physical diagnosis, founded on the presumed isolation of disease, must be used with great caution. I cannot, even at the risk of being charged with understating the position of physical investigation at the present day, avoid expressing my opinion that a too great positiveness marks some of the statements in our standard works, and that the difficulties of special diagnosis are still infinitely greater than many might be led to believe."

The same view is reiterated in the following remarks: "I desire also to enter a protest against the tendency, still too prevalent in many schools, which would base the diagnosis of disease in great part, if not entirely, on the consideration of purely physical signs, to the exclusion of that important class of phenomena, which, for want of a better name, we are obliged still to call vital. For there is nothing more calculated than this to cause the neglect of that first and greatest lesson in medicine, which, while inculcating modesty and caution in diagnosis, makes us bring every possible light to bear on the case before us.

"As the student, fresh from the schools, and proud of his supposed superiority in the refinements of diagnosis, advances into the stern realities of practice, he will be taught greater modesty and a more wholesome caution; he will find, especially in chronic disease, that important changes may exist without corresponding physical signs,—that as disease advances, its original special evidences may disappear,—that the signs of a recent and trivial affection at one portion of the heart may altogether obscure or prevent those of a disease longer in standing and much more important,—that functional alteration may not only cause the signs of organic lesion to vary infinitely, but even to wholly disappear, —that the signs on which he has formed his opinions to-day may be wanting to-morrow,—and lastly, that to settle the simple question between the existence of functional and that of organic disease, will occasionally baffle the powers of even the most enlightened and experienced physician."

The book contains twelve chapters. In these are respectively considered, Inflammation of the Heart and its Membranes—Diseases of the Valves—of the Muscular Structures—Weakness, or deficient muscular power—Fatty degeneration—Treatment of the Organic Diseases of the Heart—of the Condition of the Heart in Typhus Fever—of Displacement—of Rupture—of Deranged Action—Aneurism of the Thoracic Aorta—of the Abdominal Aorta.

The following exhibits Dr. Stokes' practical character of mind:

"We should by no means underrate the importance of differential diagnosis in valvular disease; but the number of cases in which it is desirable to determine the exact seat and nature of the affection is comparatively small. Let us take the two most ordinary forms of this disease, namely, the insufficiency, with contraction on the one hand, and dilatation on the other, of the mitral and aortic valves. Certain rules of treatment are supposed applicable to each of these affections; but the truth is, that no constant state of the heart's muscles is attendant on them respectively, and it is mainly on the vital and mechanical conditions of the cavities of the heart that we can found any rule of treatment."

"It will not be out of place to remark, that sudden death in disease of the heart is by no means so frequent as is generally supposed. In the majority of cases, death occurs in no sudden or extraordinary manner. It is principally in examples of solutions of continuity, such as the rupture of an aneurism, the laceration of the ventricles, or the breaking of the chordæ tendineæ, that this happens. We may add to this list a few cases of the fatty degeneration of the heart in which, without rupture, death takes place by a sudden syncope or a congestive apoplexy. But these are the exceptions, and in the greater proportion sufficient notice is given of the approach of death by long-continued symptoms of dropsy, and of pulmonary and hepatic disease."

Each affection of the heart is illustrated with appropriate cases, in which besides its physical signs, we have given us the general symptoms and complication of the disease. These cases are perfect models in their way, both as regards the manner in which they are drawn up, and the clear and happy style in which they are written. Few writers possess Dr. Stokes' ability of clear expression and illustration, undeformed by either prolixity or too great conciseness. All must acknowledge that there is no jewel which may not be either greatly marred or enhanced in beauty by its setting.

We most heartily commend the work to the perusal of our readers.

